# ACLY Nuclear Translocation in Human Macrophages Drives Proinflammatory Gene Expression by NF-κB Acetylation

**DOI:** 10.3390/cells10112962

**Published:** 2021-10-30

**Authors:** Anna Santarsiero, Paolo Convertini, Simona Todisco, Ciro L. Pierri, Anna De Grassi, Niamh C. Williams, Dominga Iacobazzi, Giulio De Stefano, Luke A. J. O’Neill, Vittoria Infantino

**Affiliations:** 1 Department of Science, University of Basilicata, 85100 Potenza, Italy; anna.santarsiero@unibas.it (A.S.); paolo.convertini@gmail.com (P.C.); simona.todisco@unibas.it (S.T.); 2 Department of Biosciences, Biotechnology and Biopharmaceutics, University of Bari, Via E. Orabona 4, 70125 Bari, Italy; ciro.pierri@uniba.it (C.L.P.); anna.degrassi@uniba.it (A.D.G.); 3 Trinity Biomedical Sciences Institute, School of Biochemistry and Immunology, Trinity College Dublin, 152-160 Pearse Street, Dublin 2, D02 R590 Dublin, Ireland; willianc@tcd.ie; 4 Bristol Heart Institute, Bristol Medical School, University of Bristol, Bristol BS2 8HW, UK; domingaiacobazzi@live.it; 5 Department of Infectious Diseases, San Carlo Hospital, Via Potito Petrone, 85100 Potenza, Italy; drgiuliodestefano@gmail.com

**Keywords:** ACLY, inflammation, macrophages, nuclear translocation, NF-κB, p65 acetylation, immunometabolism, gene expression, sepsis, PAMPs

## Abstract

Macrophage stimulation by pathogen-associated molecular patterns (PAMPs) like lipopolysaccharide (LPS) or lipoteichoic acid (LTA) drives a proinflammatory phenotype and induces a metabolic reprogramming to sustain the cell’s function. Nevertheless, the relationship between metabolic shifts and gene expression remains poorly explored. In this context, the metabolic enzyme ATP citrate lyase (ACLY), the producer of citrate-derived acetyl-coenzyme A (CoA), plays a critical role in supporting a proinflammatory response. Through immunocytochemistry and cytosol–nucleus fractionation, we found a short-term ACLY nuclear translocation. Protein immunoprecipitation unveiled the role of nuclear ACLY in NF-κB acetylation and in turn its full activation in human PBMC-derived macrophages. Notably, sepsis in the early hyperinflammatory phase triggers ACLY-mediated NF-κB acetylation. The ACLY/NF-κB axis increases the expression levels of proinflammatory genes, including *SLC25A1*—which encodes the mitochondrial citrate carrier—and *ACLY*, thus promoting the existence of a proinflammatory loop involving *SLC25A1* and *ACLY* genes.

## 1. Introduction

Post-translational modifications (PTMs) of proteins regulate a wide range of their biological functions such as gene expression and protein localization and activity. Acetylation of lysine residues is among the best-studied PTMs, especially relating to histone proteins, which need acetyl-CoA as an acetyl group donor. Several studies in yeast as well as in mammalian cells have highlighted the link between intracellular acetyl-CoA levels and protein acetylation [1]. Thus, protein acetylation may represent a fast way to control protein activities through metabolic changes such as acetyl-CoA fluctuations.

In mammals, acetyl-CoA is mainly supplied by ATP citrate lyase (ACLY) activity [2], when the tricarboxylic acid (TCA) cycle of intermediate citrate—generated either from glucose or glutamine—moves from the mitochondria to the cytosol through the mitochondrial citrate carrier (CIC) encoded by the *SLC25A1* gene. Mitochondrial citrate export leads to the production of acetyl-CoA and oxaloacetate via ACLY.

In murine bone marrow-derived macrophages, increased glycolysis and TCA cycle rewiring promote citrate synthesis within 2 h after lipopolysaccharide (LPS) stimulation. Meanwhile, ACLY is activated by phosphorylation since its function is required for gene expression pattern reprogramming [3]. Of note, M1 macrophages and activated natural killer cells show great ACLY upregulation as part of their own metabolic reprogramming [4,5].

In silico analysis of the human ACLY gene promoter revealed an active nuclear factor-κB (NF-κB) response element by which ACLY is upregulated upon LPS, as well as tumor necrosis factor α (TNFα) treatment of macrophages [6].

NF-κB is a family of transcription factors encompassing five members which control transcriptional regulation of the target genes as hetero- or homodimers [7]. In the absence of inducers, NF-κB is kept inactive in the cytoplasm. Following the activation of pattern recognition receptors (PRRs) or TNF receptors (TNFRs), the canonical NF-κB pathway induces a fast and transient translocation to the nucleus, mostly of p50/p65 (RelA) dimer, where it promotes the upregulation of pro-inflammatory genes in innate immune cells [8,9]. Once in the nucleus, reversible acetylation of p65—mainly to lysines 218, 221, and 310—regulates NF-κB function. Lysines 218 and 221 are highly conserved in all mammalian Rel proteins, and their acetylation affects DNA binding and assembly with IkBalpha. Conversely, lysine 310 is a specific amino acid of p65, and its acetylation is essential for the complete activation of NF-κB and transcriptional activity [10], while p65 represents a novel nonhistone substrate of histone deacetylase 3 (HDAC3), as its presence abolishes p65 acetylation. Of note, HDAC3 has a crucial role in regulating innate immune cell functions, since it is required for the inflammatory gene expression program in macrophages [11]. Nevertheless, little is known about the relationship between NF-κB and metabolic reprogramming in immune cells.

As a consequence of late ACLY transcriptional upregulation, M1 macrophages produce critical inflammatory mediators such as nitric oxide (NO) and prostaglandin E_2_ (PGE_2_), whose levels are reduced in the presence of ACLY inhibitors or gene silencing [5].

Given the importance of controlling various nuclear functions, ACLY can also translocate to the nucleus to supply acetyl-CoA and participate in cellular differentiation as well as DNA repair by homologous recombination [12]. However, no information about ACLY localization in macrophages has been published. Moreover, the molecular events linking the early and late ACLY activation are unknown.

Here, we report a timely ACLY translocation from the cytosol to the nucleus, where its function is required not only for histone but also for p65 acetylation in human LPS-induced macrophages. Furthermore, we observed that the same signaling was triggered by other PAMPs like lipoteichoic acid (LTA), pointing out ACLY mediated-p65 acetylation as a more general mechanism in macrophage activation. We also investigated the ACLY/NF-κB axis in patients with sepsis in the early hyperinflammatory phase. ACLY/NF-κB signaling is important to drive the inflammatory response through both gene expression and metabolic reprogramming. Indeed, NF-κB full activation, beyond enhancing the transcription of proinflammatory genes like *IL-1β* and *PTGS2,* allows the activation of the mitochondrial citrate export pathway by *ACLY* and *SLC25A1* transcriptional upregulation, thus fostering the inflammatory response.

## 2. Materials and Methods

### 2.1. Isolation and Differentiation of Human Monocytes

Primary human monocytes were isolated from the blood of healthy donors or patients with sepsis in agreement with the Declaration of Helsinki and in accordance with the local Italian Committee on Human Research’s approved procedures (REF. TS/CEUR 20200034750—15 September 2020). The volunteers provided written, informed consent, approving and authorizing the use of their material for research purposes. Whole venous blood was collected, and peripheral blood mononuclear cells (PBMCs) were separated by Histopaque density gradient centrifugation as reported [13]. The PBMCs were incubated with CD14 antibody conjugated to magnetic beads (MACS^®^, Miltenyi Biotec GmbH, Bergisch Gladbach, Germany). Isolated CD14+ monocytes were differentiated to macrophages by culturing in complete RPMI 1640 medium supplemented with 10 μg/mL of recombinant human M-CSF (Cell Guidance Systems, St. Louis, MO, USA) for 3 days.

### 2.2. Cell Culture and Treatments

Immortalized bone marrow-derived macrophage cells (iBMDM cells [14]) were grown in complete Dulbecco’s Modified Eagle Medium (DMEM, Thermo Fisher Scientific, Waltham, MA, USA). Lipopolysaccharide (1 µg/mL) from *E. coli* (LPS, AdipoGen Life Sciences, Inc., San Diego, USA) was added to the iBMDM cells and human macrophages for the time points described in the figure legends. Human macrophages were treated with 100 µg/mL lipoteichoic acid from *Staphylococcus aureus* (LTA, Sigma-Aldrich, St Louis, MO, USA). Where indicated, the cells were treated with 5 μM SB-204990 (a gift from GlaxoSmithKline, Brentford, UK) or 2 μg/mL actinomycin D (Sigma-Aldrich).

### 2.3. Collection of Human Septic Samples

Anonymized human whole venous blood samples from patients with sepsis caused by bacterial infections and in the early hyperinflammatory phase were obtained from the Department of Infectious Disease of San Carlo Hospital in Potenza, Italy (Table 1). Samples were collected from May 2021 to July 2021. All the cases satisfied the sepsis definition of the 2016 task force from the Society of Critical Care Medicine and European Society of Intensive Care Medicine (SCCM/ESICM) of life-threatening organ dysfunction caused by a dysregulated host response to infection [15]. Organ dysfunction is defined as an increase of two or more points in the sequential (sepsis-related) organ failure assessment (SOFA) score. The markers that were constantly present were leukocytosis, elevated plasma C-reactive protein and procalcitonin, hyperlactatemia, thrombocytopenia, and acute oliguria (Table 1).

### 2.4. Quantitative Real-Time PCR

The total RNA was extracted from 2 × 10^6^ human PBMC-derived macrophages triggered by LPS using the RNeasy Plus Mini Kit (Qiagen, Hilden, Germany) according to the manufacturer’s instructions. Complementary DNA was obtained by a GeneAmp™ RNA PCR Core Kit (Thermo Fisher Scientific) with random hexamers and murine leukemia virus reverse transcriptase (15 min at 42 °C and 5 min at 99 °C). Real-time PCR experiments were performed in triplicate with ACLY (Hs00982738, RefSeq NM_001096.2) and *β*-actin (Hs01060665, RefSeq NM_001101.3) TaqMan Gene Expression Assays (Thermo Fisher Scientific) on a 7500 Fast Real-Time PCR System (Thermo Fisher Scientific). *β*-Actin was used as an endogenous reference gene for normalization of the ACLY expression levels. To this end, the *β*-actin Ct value was subtracted from the ACLY Ct value, generating a ΔCt value. ΔΔCt was calculated by subtracting the mean value of ΔCt of the untreated cells from the ΔCt of the LPS-triggered cells. Finally, the fold changes in ACLY gene expression were calculated via the comparative 2^−ΔΔCt^ method [16].

### 2.5. Western Blotting

The cells (1 × 10^6^) were suspended in a lysis buffer (50 mM Tris-HCl pH 8.0, 150 mM NaCl, 0.5% sodium deoxycholate, 0.1% sodium dodecyl sulfate, 1 mM EDTA, 1% Nonidet P-40, and 1× protease inhibitors cocktail), mechanically lysed 10 times in a 1-mL syringe, and put in a rotating shaker for 30 min at 4 °C. After centrifugation at 8600× *g* for 5 min at 4 °C, the supernatant was collected, and the protein concentration was determined by a Bradford assay. A Laemmli sample buffer was added to 30 micrograms of proteins. The samples were boiled at 100 °C for 5 min, subjected to SDS-PAGE, and then electroblotted onto nitrocellulose membranes. The membranes were blocked for 1 h in a tris-buffered saline (TBS) solution containing 5% non-fat dry milk and 0.5% Tween and then immunostained with anti-ATP citrate lyase, anti-NF-κB/p65, anti-acetylated H3, anti-total H3, anti-IL-1β, anti-cyclooxygenase-2 (COX2), anti-acetylated lysine, anti-AcK310 NF-κB/p65, anti-Lamin, anti-citrate carrier (CIC), or anti-β–actin primary antibodies. Almost all the antibodies were purchased from Abcam (Cambridge, MA, USA) except the anti-acetylated lysine (Cell Signaling Technology, Danvers, MA) and anti-CIC (Thermo Fisher Scientific), as reported in Table S1. Following incubation with a horseradish peroxidase (HRP) goat anti-rabbit IgG secondary antibody, the immunoreactions were detected by using the HRP substrate WesternBright™ ECL (Abcam) in a Chemidoc™ XRS detection system equipped with Image Lab software for image acquisition and densitometric analysis (Bio-Rad Laboratories, Hercules, CA, USA).

### 2.6. Transient Transfections and RNA Interference

The iBMDM cells were transiently transfected with an ACLY wild-type (Pwt) construct, ACLY double Ala mutant (Pmut) construct obtained from Genscript Biotech (Netherlands), NF-κB reporter plasmid containing a firefly luciferase gene driven by five copies of NF-κB response element (5′-GGGACTTTCC-3′) located upstream of the minimal TATA box promoter (pGL3–5xNF-κB), pGL3 basic-LUC vector (Promega, Madison, WI, USA) containing the −1785/−20 bp region of the SLC25A1 gene promoter (SLC25A1pGL3) [17], and pGL3 basic-LUC vector containing the −3116/−20 bp region of the ACLY gene promoter (3000) or a deletion fragment of this region (1000) [18] (Table S2). Where indicated, a construct overexpressing the NF-κB p65 subunit wild-type (NF-κB wt) or mutated K310R (NF-κB mut) (Genscript Biotech) was also used in cotransfection experiments (Table S2). To normalize the extent of transfection, the cells were transfected with 10 ng of pRL-CMV (Promega). Twenty-four hours after transfection, the iBMDM cells were triggered by LPS. The day after, the cells were lysed and assayed for LUC activity using the Dual-Luciferase^®^ Reporter Assay System (Promega) according to the manufacturer’s protocol. Luminescence was measured on a GloMax 96-well luminometer from Promega. The ACLY gene was transiently silenced by RNA interference experiments transfecting iBMDM cells for two consecutive days with a specific small interfering RNA (siRNA) targeting human ACLY (Validated Silencer^®^ Select, Thermo Fisher Scientific) or control scramble siRNA (Thermo Fisher Scientific) (Table S2) using Lipofectamine RNAiMax Reagent as described previously [19].

### 2.7. Immunocytochemistry

The iBMDM cells, induced with LPS and treated as described in the figure legends, were washed in Phosphate Buffered Saline (PBS) and fixed by cross-linking with 3% paraformaldehyde solution. Following permeabilization with PBS + 0.25% Triton X-100 (PBST) and blocking with PBST + 1% bovine serum albumin (BSA), the cells were incubated with anti-ACLY or anti-DDDDK tag primary antibodies (Table S1) at 4 °C overnight. The day after, an Alexa Fluor 488 goat anti-rabbit IgG secondary antibody (A-11008, Thermo Fisher Scientific) was used while Fluoroshield Mounting Medium with DAPI (ab104139, Abcam) was employed to preserve the fluorescence and as a counterstain for DNA. The images were obtained with a fluorescence microscope (EVOS FLoid Cell Imaging Station, Thermo Fisher Scientific). The density of the immunolabeled protein was normalized to the number of DAPI positive cells (nuclear/cytosolic ratio) by using ImageJ software (https://imagej.nih.gov/ij/index.html, NIH, accessed on 28 October 2021).

### 2.8. ACLY Activity

The macrophages (5 × 10^6^) were activated with LPS. At the end of the treatments, the cells were washed twice in ice-cold PBS by centrifugation at 8600x *g* at 4 °C for 5 min. The cell pellet was resuspended in ice-cold 0.1 % Nonidet P-40 (NP-40) in PBS, and 3 freeze-melt cycles (−80 °C for 8 min and 40 °C for 4 min) were performed. After centrifugation (8600× *g* at 4 °C for 5 min), the supernatant was collected, and the protein concentration was determined by a Bradford assay. ACLY activity was assessed by the coupled malic dehydrogenase method [20]. The assay mixture was made of 150 μg of extract, 50 mM Tris-HCl (pH 8.0), 10 mM MgCl_2_, 10 mM DTT, 0.2 mM NADH (Sigma-Aldrich), 0.3 mM CoA (Sigma-Aldrich), 5 mM ATP (Sigma-Aldrich), 20 mM potassium citrate, and 5 units/mL malic dehydrogenase (10127914001, Sigma-Aldrich). The reaction was initiated by adding ATP and followed by monitoring the decrease in NADH absorbance at 340 nm at 25 °C for 20 min with a spectrophotometer (Multiskan Sky, Thermo Fisher Scientific). The specific ACLY activity was expressed as a percentage of the control.

### 2.9. Cytosol Nucleus Fractionation

The cells were collected (2 × 10^6^), and after a short centrifugation (3 min at 300× *g*) and washing with ice-cold PBS, the pellet was resuspended in an ice-cold 0.1% NP-40-PBS buffer. After pipetting up and down 10 times and centrifugation (4 °C) for 1 min at 8600× *g*, the supernatant was collected (cytosolic fraction), and the pellet was resuspended in an ice-cold 0.1% NP-40-PBS buffer. The nuclear fraction was obtained after centrifuging (4 °C) for 5 min at 8600× *g*, resuspended in 200 µL NP40 0.1%, and sonicated using microprobes at 36–40% power twice for 10 s before the western blotting experiment.

### 2.10. Co-Immunoprecipitation

The monocytes (1 × 10^7^) were seeded in 10 cm dishes in 10 mL of macrophage media, and after differentiation, the cells were treated as required. At the end of the treatment, the cells were washed with PBS before lysis in a 1 mL cold low-stringency lysis buffer (100mM NaCl, 10% glycerol, 50 mM HEPES pH 7.5, 1mM EDTA, 0.5% NP-40, 10 μg/mL aprotinin, 1mM phenylmethylsulfonyl fluoride (PMSF), 1 μg/mL leupeptin, and 1 mM sodium orthovanadate). The dishes were scraped with a scraper, and the cell lysate was kept on ice for 15 min before centrifugation at 12,000× *g* for 10 min at 4 °C.

Anti-ATP citrate lyase, anti-acetyl lysine, or anti-NF-κB/p65 primary antibodies (Table S1) and 40 μL A/G PLUS agarose beads (SC-2003, Santa Cruz Biotechnology, Santa Cruz, CA, USA) were added to the supernatant, which was then incubated at 4 °C on a rocking platform for 2 h and centrifuged at 400× *g* for 2 min at 4 °C. Subsequently, the supernatant was removed, and the beads were washed three times with a 1-mL low stringency lysis buffer. Finally, the immune complexes were eluted with a 50-μL Laemmli sample buffer, boiled for 5 min, and analyzed by western blotting.

### 2.11. ChIP-qPCR and ReChIP-qPCR

The chromatin immunoprecipitation (ChIP) experiments were performed as reported previously [21]. The cells (5 × 10^6^) were treated with LPS in the presence or absence of SB for 3 h. To investigate the H3 acetylation levels, a native ChIP experiment was performed. The cells were lysed in a 0.1% NP-40-PBS buffer plus protease inhibitor cocktail (PIC), put on ice for 10 min, and centrifuged at 1000 × *g* for 2 min at 4 °C. The pellet was suspended in a 0.1% NP-40-PBS buffer and sonicated at 70% power at cycle 9 for 25 min to have bands of 500–1000 bp. The chromatin (20 µg) was immunoprecipitated in an incubation buffer (10 mM Tris-HCl pH8; 150 mM NaCl; 10 mM KCl; 1 mM EDTA; 0.1% NP-40) plus 30 µL of Dynabeads Protein G (Thermo Fisher Scientific) overnight on rocking platform with an anti-histone H3 (acetyl K9 + K14 + K18 + K23 + K27) antibody (Table S1). The day after, the input (total chromatin extract) and mock (immunoprecipitation without the antibody) samples were recovered and then used for qPCR analysis. The protein/DNA complexes were retained and washed with PBS. After treatment with RNase and Protease K, the DNA was purified by using a PureLink™ PCR Purification Kit (Thermo Fisher Scientific) and analyzed by qPCR using the primers reported below.

For immunoprecipitation with the anti-NF-κB/p65 antibody, following the required treatments, the cells were fixed by 1% formaldehyde at 37 °C for 10 min. Afterward, the cells were lysed and sheared by sonication at 70% power at cycle 9 for 10 min in a 0.1% SDS lysis buffer to generate cellular chromatin fragments of 300–400 bp. The chromatin was immunoprecipitated overnight at 4 °C on a rocking platform using 40 µL A/G PLUS agarose beads (SC-2003, Santa Cruz) with anti-NF-κB/p65- or anti-NF-κB/p50-specific antibodies (Table S1).

In the Re-ChIP experiments, after chromatin immunoprecipitation with the anti-NF-κB/p50 antibody, the samples were subjected to a second immunoprecipitation with anti-AcK310 NF-κB/p65 (Table S1). The input and mock samples were recovered, while the protein/DNA complexes were retained and washed by using an RIPA buffer (10 mM Tris-HCl, pH 8.0, 1 mM EDTA, 0.5 mM EGTA, 1% Triton X-100, 0.1% sodium deoxycholate, 0.1% SDS, 140 mM NaCl, and 1 mM PMSF), LiCl Buffer (0.25 M LiCl, 0.5% NP-40, 0.5% deoxycholic acid, 10 mM TrisHCl pH 8.0, 1 mM EDTA) and a Tris-EDTA buffer (10 mM Tris-HCl, 1 mM disodium EDTA, and pH 8.0). After reverse cross-linking with Protease K, the DNA was purified and analyzed by qPCR using the following oligonucleotides: ForPTGS2 5′-CCGCTTCCTTTGTCCATCAG-3′; RevPTGS2 5′-TTGGAAAGAGAGGCGGGAAA-3′; ForACLY 5′-CTTTCCAAAGTTGGGTCTTGTG-3′; RevACLY 5′-CCTCAGCAATTCAGACTCCTT-3′; ForIL1β 5′-GTCTTCCACTTTGTCCCACATA-3′; RevIL1β 5′-CTGACAATCGTTGTGCAGTTG-3′; ForSLC25A1 5′-TCTGCTCATTGTGGGCTTC-3′; and RevSLC25A1 5′-GTTGGGAGCAGAGGCATATC-3′.

The 20-µL qPCR reaction was made of 200 ng of DNA, 10 µL of SYBR™ Green PCR Master Mix (Thermo Fisher Scientific), 20-pmol forward and reverse primers, and nuclease-free water. A melting curve analysis was performed upon completion of each run to check the specificity of the primers. The qPCR reactions were run on a 7500 Fast Real-Time PCR System (Thermo Fisher Scientific).

### 2.12. Mass Spectrometry Analysis

The ACLY was immunoprecipitated from 5 mg of macrophage proteins, and IP samples were separated on SDS–PAGE gels and Coomassie stained before the bands of interest were excised from the gel. Blind mass spectrometry analysis was performed by PTM Biolabs (Chicago, IL, USA) as described below. The gel pieces were digested with trypsin at 37 °C overnight. After extraction with 50% acetonitrile and 5% formic acid, the peptides were cleaned with C18 ZipTips (Merck Millipore, Darmstadt, Germany). Then, the peptides were subjected to a nanospray ion source followed by tandem mass spectrometry (MS/MS) using the Q Exactive™ (Thermo Fisher Scientific). The resulting MS/MS data were processed using the Mascot search engine (v.2.3.0, Matrix Science, London, UK). Carbamidomethylation on the cysteines was specified as a fixed modification. Acetylation on the lysine was specified as variable modification.

### 2.13. Citrate Lyase 3D Comparative Analyses

The sequence of the human citrate lyase NP_001290203.1 was used as a query sequence for running pGenThreader and I-TASSER web services for identifying putative crystallized structures for the following structural analyses. Notably, both I-TASSER and pGenThreader suggested a group of two “high-confidence” putative template structures of the human citrate lyase crystallized complex with citrate, ADP, and 2 Mg^2+^ ions (5tes.pdb) [22,23] and with CoA (6hxh.pdb) [24] to be used for the following structural analysis. Starting from the two cited crystallized structures, it was possible to superimpose them by using PyMOL (through the “align” and “super” tools implemented in the software) and creating a unique 3D model structure hosting the citrate substrate and the cited cofactors, allowing the study of the localization of the two investigated residues (K662 and K665, NP_001290203.1 numbering) with respect to the localization of the citrate, ADP, and CoA binding region according to the protocols described in [22,23,24] and the references therein. The resulting final 3D structure corresponded to the 3D structure of chain A of the 6hxh.pdb structure that also hosted a CoA ligand (in spite of 5tes.pdb) after having verified that the coordinates of the citrate, ADP and Mg^2+^ did not change by superimposition with 5tes.pdb. The acetyl groups were added to the investigated K662 and K665 residues by using the “fragment” addition tool implemented in PyMOL. The resulting structure was energetically minimized by using the Yasara “energy minimization server” (http://www.yasara.org/minimizationserver.htm, accessed on 28 October 2021) [25]. The images of the provided 3D model coordinates were created with PyMOL (https://pymol.org/2/, accessed on 28 October 2021).

### 2.14. Quantification and Statistical Analysis

Statistical analyses were performed using the statistics tools implemented in the GraphPad Prism software. The results are represented as the mean ± standard deviation (SD) from at least three independent experiments, each run in triplicate. For pairwise comparisons, a Mann–Whitney U test was performed. Comparisons of more than two groups were evaluated using one-way ANOVA followed by Dunnett’s or Tukey’s multiple comparison tests. The statistical methods used for each experiment are detailed in the figure legends. The asterisks in the figures denote statistical significance (* *p* < 0.05; ** *p* < 0.01; and *** *p* < 0.001). When Tukey’s post hoc test was performed, different letters indicated significant differences between treatments at *p* < 0.05.

## 3. Results

### 3.1. Early ACLY Expression and Activity in LPS-Triggered Human Macrophages

**Figure 1:**
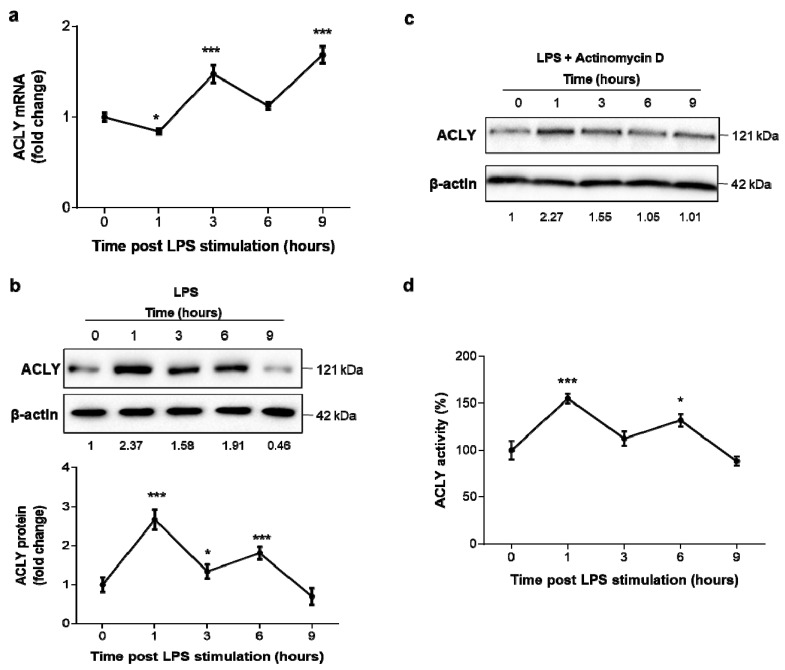
Early ACLY expression and activity in LPS-triggered human macrophages. Human PBMC-derived macrophages were triggered by LPS, and real time PCR and western blot analyses were performed to evaluate the ACLY mRNA (**a**) and protein (**b**) levels, respectively. The bottom panel in (**b**) shows ACLY protein quantitation across 6 independent western blots. (**c**) ACLY and β-actin proteins from human PBMC-derived macrophages, treated as described above but in the presence of actinomycin D, were detected by specific antibodies. (**d**) Time course of ACLY activity in human PBMC-derived macrophages. In (**a**,**d**), data are representative of 3 independent experiments and are presented as means ± SD (error bars). Where indicated in (**a**,**d**), differences were significant according to one-way ANOVA followed by Dunnett’s multiple comparison test (* *p* < 0.05; and *** *p* < 0.001). The mean values of treated cells were normalized to the mean values of untreated cells. Western blotting data presented are representative of at least 3 independent experiments. Protein levels quantified against β-actin and normalized versus untreated cells (0) are reported under western blotting images.

To better understand the early involvement of ACLY in LPS-induced macrophages, we performed a gene expression time course in human PBMC-derived macrophages. The real-time PCR experiments showed an ACLY mRNA increment at 3 h after LPS’s addition (Figure 1a). Conversely, by analyzing the ACLY protein over the same time course, early upregulation was observed within 1 h after LPS stimulation. At this time point (1 h post LPS), a doubled amount of ACLY protein was found, followed by a decline at 3 h post LPS addition (Figure 1b). Six hours after LPS treatment, a second ACLY increase was present, followed by a rapid decrease (Figure 1b). These results revealed a rise in the ACLY protein content first (1 h after LPS addition; Figure 1b) before its transcriptional activation (i.e., earlier than the first mRNA peak observed 3 h after LPS stimulation (Figure 1a)). Therefore, we thought that the early increase in ACLY protein, preceding the ACLY mRNA increment, could be non-transcriptionally regulated, as it was not subsequent to an mRNA peak.

To gain more insight into the molecular mechanisms underlying ACLY activation in LPS-triggered inflammation, we next determined the time-dependent ACLY protein expression profile under the same conditions described above but in the presence of actinomycin D, a strong inhibitor of transcription. As illustrated in Figure 1c, actinomycin D did not affect the at first increased amount of ACLY protein observed 1 h after LPS’s addition in Figure 1b. Conversely, the second peak 6 h after treatment (shown in Figure 1b) was transcription-dependent, since it disappeared when the transcription was inhibited (Figure 1c). Hence, these data indicate that de novo transcription is not required for the first ACLY response but only for its late activation, suggesting a possible posttranscriptional regulation as previously reported in rats [26]. Therefore, to define the function of ACLY in human LPS-activated macrophages, we evaluated ACLY activity at different times. The activity trend reflected the protein concentration levels, showing two peaks at 1 h and 6 h after LPS treatment (Figure 1d). These findings reveal that LPS induces a fast non-transcriptional activation and then a transcriptional upregulation of ACLY.

### 3.2. ACLY Translocates from the Cytosol to the Nucleus after LPS Treatment

**Figure 2:**
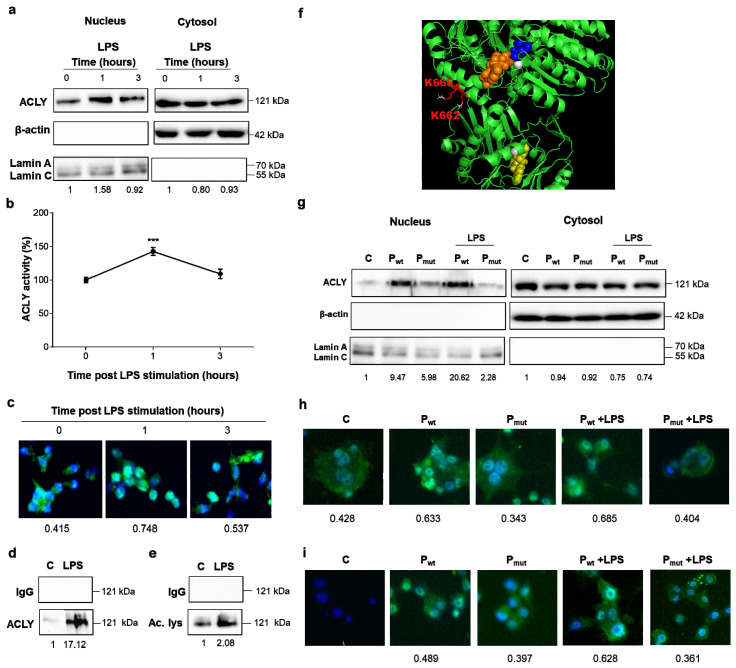
LPS induced ACLY acetylation and, in turn, its nuclear translocation. Human PBMC-derived macrophages were triggered by LPS, and (**a**) nuclei were isolated by cell fractionation. In nuclear and cytoplasm fractions, ACLY, β-actin, and lamin proteins were detected by specific antibodies. (**b**) Nuclear ACLY activity. (**c**) Time course of ACLY nuclear translocation by immunocytochemistry (ICC) using anti-ACLY and DAPI nuclear staining. (**d**) Immunoprecipitated with an antibody directed to acetylated lysine and analyzed by western blot with an anti-ACLY antibody. (**e**) In macrophages triggered by LPS, ACLY was immunoprecipitated with a specific antibody, and then an antibody against acetylated lysine (Ac. Lys.) was used in western blotting experiments. (**f**) A 3D model of ACLY protein based on 6hxh.pdb is reported in a green cartoon representation and in a complex with citrate (blue spheres), ADP (yellow spheres), Mg (white spheres), and CoA (orange spheres). K662 and K665 acetylated lysines are reported in red sticks. (Acetyl groups added by PyMOL on K662 and K665 are reported in white sticks) (**g**) iBMDM cells were transfected with a construct overexpressing the ACLY wild type (Pwt) and a construct overexpressing a double Ala mutant (Pmut) and used for isolating nuclei. In nuclear and cytoplasm fractions, ACLY, β-actin, and lamin proteins were detected by specific antibodies. (**h**) iBMDM cells, transfected as in (**g**), were used for ICC analysis with anti-ACLY and DAPI nuclear staining. (**i**) iBMDM cells, transfected as in (**g**), were used for ICC analysis with an anti-DDDDK tag and DAPI nuclear staining. In (**a**,**b**), IgG is the negative control. In (**b**), data are representative of 3 independent experiments and are presented as means ± SD (error bars). Statistical significance of the differences was evaluated by using one-way ANOVA followed by Dunnett’s multiple comparison test (*** *p* < 0.001). Western blotting and ICC data presented are representative of at least 3 independent experiments. In (**a**,**g**), protein levels are quantified against β-actin or lamin. In (**a**,**d**,**e**,**g**), they are normalized versus the mean of proteins in untreated cells (C) and reported under each image. Quantitation of ACLY in (**c**,**h**,**i**) ICC images was normalized to a DAPI nuclear stain.

Since ACLY may localize to both the nucleus and cytoplasm, we wondered if LPS might induce its nuclear translocation and in turn promote gene expression reprogramming. Thus, macrophages differentiated from human PBMCs and treated with LPS were used for separating the nuclear and cytoplasmic fractions. Western blotting analysis on both fractions revealed an increased amount of ACLY in the nucleus at 1 h after LPS stimulation (Figure 2a). Surprisingly, following 3 h of incubation with LPS, the nuclear ACLY was greatly reduced (Figure 2a). The purity and abundance of the nuclear fraction and the cytosolic counterpart were assessed by anti-lamin, a marker of the nuclear compartment, and β-actin antibodies, respectively. ACLY activity in the nuclear fraction confirmed early increased activity within 1 h following LPS treatment, while 3 h of LPS incubation brought ACLY activity back to the levels observed before macrophage activation (Figure 2b). The temporary stay of ACLY in the nucleus was also proven by fluorescence microscopy analysis, showing remarkable ACLY nuclear translocation only 1 h after LPS’s addition (Figure 2c).

When looking for a mechanism controlling ACLY nuclear translocation, we hypothesized that posttranslational modifications might occur in LPS-activated macrophages. By immunoprecipitation (IP) with an anti-acetylated lysine antibody, we found a great amount of acetylated ACLY following 1 h of LPS treatment (Figure 2d). To confirm these results, LPS-triggered macrophages were immunoprecipitated with anti-ACLY and analyzed with an antibody directed to acetylated lysine. Figure 2e shows a strong increase in acetylated ACLY upon LPS treatment. Mass spectrometry analysis was performed on human PBMC-derived macrophages treated for 1, 3, and 6 h with LPS to identify the putative acetylation sites. This analysis revealed the absence of acetylated lysine residues in both PBMC-derived macrophages that were untreated as well as 3 h post LPS addition. We also observed the presence of two acetylated lysine residues, K662 and K665 (NP_001290203.1 numbering), 1 h and 6 h post LPS treatment (Figure 2f and Figure S1a). K662 is evolutionarily conserved, and K665 is present in all mammals (Figure S1b). The two lysine residues were located at the interface between the subunit hosting the ADP cofactor and the subunit hosting the citrate and CoA substrates (Figure 2f). In addition, K662 and K665 were next to the predicted nuclear localization signal (Figure S2).

To test if ACLY nuclear translocation required acetylation of K662 and K665, we generated a wild-type construct overexpressing ACLY (Pwt) and a double Ala mutant (Pmut) with double substitution of K662 and K665 and used both constructs to transfect the iBMDM cells. Transfected cells treated with LPS, and from which the nuclei were purified, revealed a nuclear translocation of Pwt greater than Pmut (Figure 2g). The purity of the nuclear fractions was evaluated by immunoblotting lamin and by testing the absence of β-actin.

Through immunocytochemistry analysis with both anti-ACLY and anti-DDDDK tag antibodies, we confirmed the presence of Pwt in the nucleus (Figure 2h,i). The differences between 2h and 2i were possibly caused by the different antibodies used. In fact, the use of an antibody against ACLY detected not only the protein produced by the plasmids but also the endogenous ACLY (Figure 2h). The anti-DDDDK tag antibody was useful to have a signal referable only to the ACLY expressed by the plasmids (Figure 2i). All together, these findings indicate that LPS induces an early nuclear translocation of ACLY regulated by its acetylation status.

### 3.3. Nuclear ACLY Fosters NF-κB Acetylation and Its Activity

**Figure 3:**
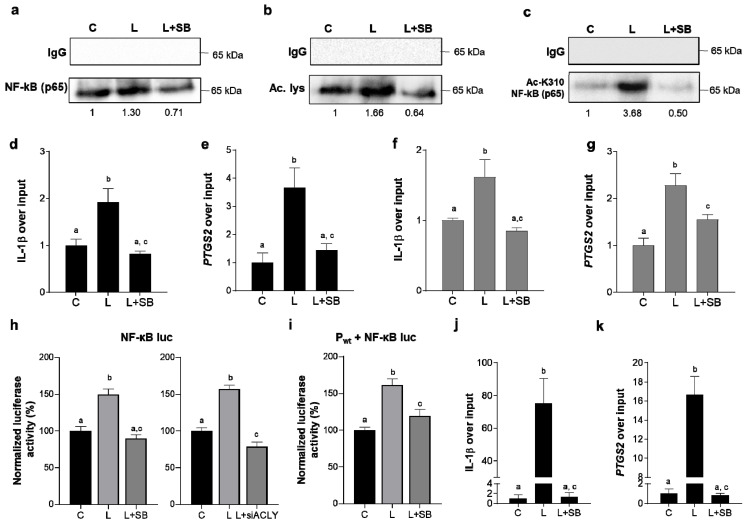
Nuclear ACLY fostered NF-κB acetylation and its activity. Human PBMC-derived macrophages were triggered by LPS in the presence or absence of SB and (**a**) immunoprecipitated with an antibody directed to acetylated lysine and analyzed by western blot with an anti-NF-κB (p65) antibody. (**b**) In macrophages triggered by LPS, with or without SB, NF-κB (p65) was immunoprecipitated with a specific antibody, and then an antibody against acetylated lysine was used in western blot experiments. (**c**) Macrophages treated and immunoprecipitated as in (**b**) were analyzed by western blot with an anti-NF-κB p65 (acetyl K310) antibody. In (**a**–**c**), IgG is the negative control. (**d**,**e**) Human PBMC-derived macrophages, treated as in (**a**), were used to carry out chromatin immunoprecipitation (ChIP) analysis with an antibody against subunit p65 of NF-κB, and then specific primers were used in the real-time PCR experiments to analyze the IL-1β and *PTGS2* gene promoters. (**f**,**g**) Human PBMC-derived macrophages, treated as reported above, were used to carry out ReChIP experiments with a first antibody against subunit p50 of NF-κB and then with an anti-NF-κB p65 (acetyl K310) antibody. Specific primers were used in the real-time PCR experiments to analyze the IL-1β and COX2 gene promoters. (**h**) Luciferase activity was quantified in iBMDM cells transiently transfected with NF-κB luc in the presence or absence of SB (left panel) or with siRNA targeting human ACLY (siACLY) or control scramble siRNA (right panel) and triggered by LPS. (**i**) iBMDM cells transfected with NF-κB luc plus an ACLY overexpression vector (P_wt_) and triggered by LPS in the presence or absence of SB were used for luciferase luminescence detection. (**j**,**k**) Human PBMC-derived macrophages treated with LPS for 3 h in the presence or lack of SB-204990 (SB) were used to carry out chromatin immunoprecipitation (ChIP) analysis with an antibody against acetylated histone H3, and then specific primers were used in the real-time PCR experiments to analyze the *IL-1β* and *PTGS2* gene promoters. In (**d**–**k**), data are representative of 3 independent experiments and are presented as means ± SD (error bars). Different letters above the bars indicate significant differences between treatments at *p* < 0.05, according to Tukey’s post hoc test performed after one-way ANOVA. Western blotting data presented are representative of at least 3 independent experiments, and the mean of protein levels normalized versus mean of proteins in untreated cells (C) is reported under each image.

It is well known that the heterodimer consisting of p65 and p50 subunits is the most abundant and the main form of NF-κB involved in inflammation [27]. Since it has been reported that lysine 310 acetylation is essential for the full transcriptional activity of p65 [28], we sought to determine if ACLY nuclear activity—apart from being required for histone acetylation—could meanwhile affect p65 acetylation. Therefore, human PBMC-derived macrophages were stimulated with LPS in the presence or absence of SB-204990 (SB), a specific ACLY inhibitor [29], after verifying that SB did not affect the cell viability (Figure S3a). Then, the cells were immunoprecipitated with an antibody directed to acetylated lysine. Western blotting analysis with an anti-NF-κB (p65) antibody showed a smaller band when SB was used compared with LPS treatment alone (Figure 3a). Similar results were obtained when performing immunoprecipitation with a specific antibody against p65 and immunoblotting with an antibody against acetylated lysine (Figure 3b). To strengthen our outcomes, macrophages immunoprecipitated with a specific antibody against p65 were analyzed by western blotting with an anti-AcK310 NF-κB (p65) antibody. As depicted in Figure 3c, LPS strongly increased K310 acetylation, which was completely abolished in the presence of SB. Interestingly, when we tested if SB affected the p65 expression levels, we found a slight reduction at the same time tested above (Figure S3b). Taken together, these findings are consistent with our hypothesis that nuclear ACLY-derived acetyl-CoA may promote the acetylation not only of histone proteins but also of p65 in order to support proinflammatory gene expression. Following the results described above, we wondered if ACLY-mediated p65 acetylation may affect NF-κB binding to its target gene promoters. To this end, we immunoprecipitated with an anti-p65 antibody the chromatin of *IL-1β* and *PTGS2* proinflammatory genes controlled by NF-κB [30,31]. ChIP analysis revealed a decreased binding to both promoters when ACLY activity was inhibited (Figure 3d,e). This result, while being important, does not provide a complete explanation for what we had assumed. However, a such specific antibody like anti-AcK310 p65, which is critical to prove that ACLY-mediated p65 acetylation affects NF-κB binding to its target promoters, could not be used directly on all chromatin, because it needs to work in an environment “enriched” with NF-κB to recognize acetylated p65. Hence, we performed Re-ChIP experiments where we first immunoprecipitated with an anti-p50 antibody in order to create an environment “enriched” with NF-κB and then with an anti-AcK310 p65 antibody to answer our hypothesis.

Interestingly, the Re-ChIP assays unveiled that ACLY inhibition strongly reduced the binding to both the *IL-1β* and *PTGS2* gene promoters (Figure 3f,g). Therefore, Re-ChIP analysis was crucial in demonstrating that ACLY inhibition lowered NF-κB binding to the promoter by diminishing p65 acetylation.

The IL-1β and COX2 protein levels, as well as the secretion of IL-1β and PGE_2_, were also reduced in the presence of ACLY inhibition (Figure S3c,d). These data suggest that ACLY, aside from K310, likely could regulate acetylation of other p65 lysines, such as K218 and K221, which are mostly involved in DNA binding.

In addition, ACLY inhibition reduced the NF-κB transactivation efficiency in transient transfection, with a vector where the promoter driving the luciferase gene reporter encompassed five copies of the NF-κB response element (Figure 3h, left panel). The same experiment was also performed in ACLY-silenced cells. First, we measured the ACLY gene silencing efficiency to be about 80% (Figure S3e). Notably, as shown in Figure 3h (right panel), ACLY gene silencing decreased the NF-κB transactivation efficiency by about 50% compared with the unsilenced cells.

Finally, the transiently transfected Pwt ACLY overexpressing construct affected NF-kB-mediated promoter regulation, whereas Pmut had no effect (Figure 3i and Figure S3f).

Since the activity of transcriptional factors is thus intimately tied to the chromatin structure [32], we verified that ACLY-mediated histone acetylation occurred in human macrophages, too. To this end—confirming the decrease of histone H3 acetylation levels in SB-treated human macrophages (Figure S3g)—we analyzed the effect of ACLY inhibition on *IL-1β* and *PTGS2* proinflammatory gene expression through ChIP analysis by using a specific antibody against the acetylated histone H3 in the presence or absence of SB. As shown in Figure 3j,k, LPS induced a strong increase of the histone H3 acetylation levels, but SB’s addition completely abolished this effect in both the promoters analyzed. Our data so far indicated that the early ACLY-mediated NF-κB full activation through acetylation of subunit p65 was required for proinflammatory gene upregulation. Likely promoted by the nuclear ACLY activity, histone acetylation at the same gene promoters helped LPS-triggered gene expression reprogramming.

### 3.4. LTA and Sepsis in the Early Hyperinflammatory Phase Trigger ACLY-Mediated NF-κB Acetylation

**Figure 4:**
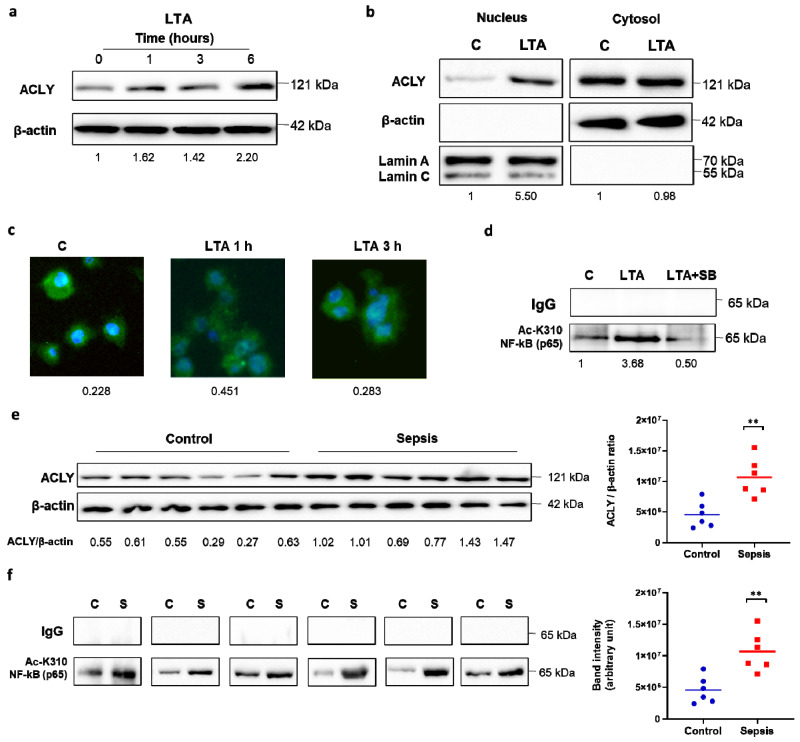
LTA and sepsis in the early hyperinflammatory phase triggered ACLY-mediated NF-κB acetylation. Human PBMC-derived macrophages were triggered by LTA, and (**a**) western blot experiments were performed to evaluate ACLY protein. (**b**) Nuclei were isolated by cell fractionation. In nuclear and cytoplasm fractions, ACLY, β-actin, and lamin proteins were detected by specific antibodies. (**c**) Time course of ACLY nuclear translocation by immunocytochemistry (ICC) using anti-ACLY and DAPI nuclear staining in iBMDM cells treated with LTA. (**d**) In human PBMC-derived macrophages triggered by LTA, with or without SB, NF-κB (p65) was immunoprecipitated with a specific antibody and then analyzed by western blotting with an anti-NF-κB p65 (acetyl K310) antibody. (**e**,**f**) Macrophages differentiated from PBMCs of patients with sepsis in the early hyperinflammatory phase and age-matched healthy controls were used to quantify ACLY protein by western blot experiments (**e**) and to immunoprecipitate NF-κB (p65) with a specific antibody and then to analyze by western blotting with an anti-NF-κB p65 (acetyl K310) antibody. (**f**) Western blotting data presented are representative of at least 3 independent experiments. In (**a**,**b**,**e**), protein levels are quantified against β-actin. In (**a**,**b**,**d**), the mean of the protein values was normalized versus the mean of the proteins in untreated cells (C), and the results are reported under each image. In (**c**), quantitation of ACLY in ICC experiments was normalized to the DAPI nuclear stain. In (**e**,**f**), statistical significance of the differences between the ACLY amount in the controls and sepsis samples evaluated by using a Mann–Whitney U test (** *p* < 0.01) is depicted in dot plots (right panels).

Considering that NF-κB activation is a hallmark of other PAMPs beyond LPS, such as Gram-positive bacterial cell wall components [33,34], we hypothesized that other PAMPs could induce ACLY-mediated NF-κB acetylation. To verify this assumption, we treated macrophages differentiated from human PBMCs with lipoteichoic acid (LTA) from *Staphylococcus aureus* and observed a significant increase in the ACLY protein levels 1 h and 6 h after LTA’s addition (Figure 4a). Moreover, ACLY moved to the nucleus upon 1 h of LTA treatment, as revealed by cytosol-nucleus fractionation analysis (Figure 4b). The detection of lamin and β-actin ensured the purity of both the nuclear and cytosolic fractions. ACLY nuclear translocation following 1 h of stimulation with LTA was confirmed by immunocytochemical experiments in the iBMDM cells using fluorescence microscopy (Figure 4c). Next, human PBMC-derived macrophages were immunoprecipitated as described in Figure 3c and immunodecorated in western blotting by an anti-AcK310 NF-κB (p65) antibody. Figure 4d depicts a great amount of p65 acetylation following LTA treatment. ACLY inhibition by SB strongly decreased p65 acetylation. Collectively, these findings support the activation of the ACLY/NF-κB axis as a general mechanism occurring in PAMP-activated macrophages.

Since NF-κB activation is increased in patients with sepsis, where the main heterodimer characterized is p50/p65 [35,36], we examined whether ACLY-mediated NF-κB acetylation is a feature of this condition. First, PBMC-derived macrophages from patients in the early acute hyperinflammatory phase of sepsis were investigated for their ACLY contents. We observed higher levels of ACLY proteins in patients with sepsis than in the age-matched healthy controls (Figure 4e). Then, p65 was immunoprecipitated, and western blotting experiments were resolved with an anti-AcK310 p65 antibody. Interestingly, NF-κB acetylation was very strong in all the patients examined, although to different extents, when compared with the healthy subjects (Figure 4f). These findings highlight the importance of the ACLY/NF-κB axis in sepsis.

### 3.5. ACLY-Mediated NF-κB Full Activation Upregulates SLC25A1 and ACLY Genes and Thus Triggers a Positive Self-Regulated Loop

**Figure 5:**
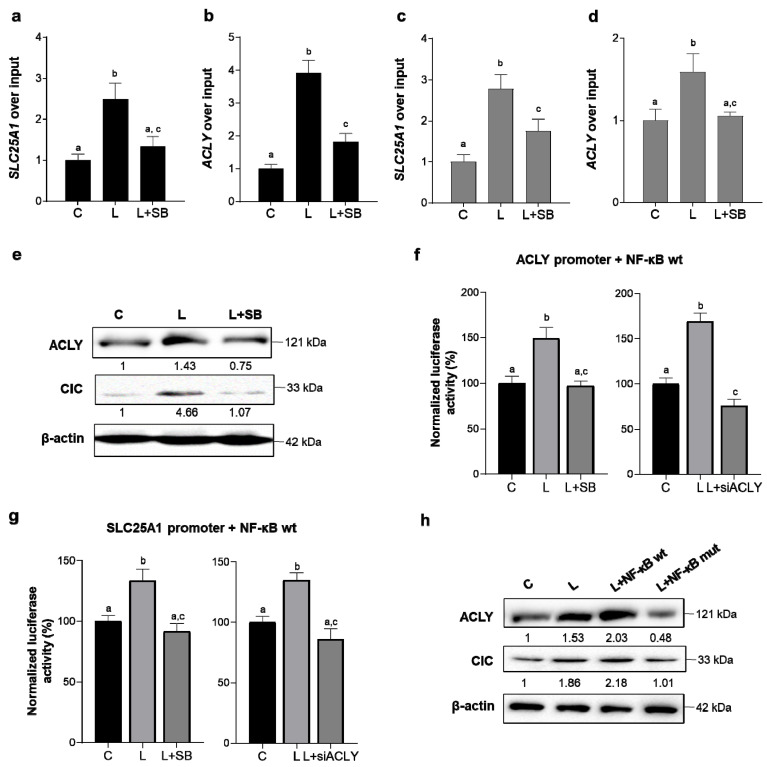
ACLY-mediated NF-κB full activation upregulated the *SLC25A1* and *ACLY* genes. Human PBMC-derived macrophages were triggered by LPS in the presence or absence of SB, and (**a**,**b**) were used to carry out chromatin immunoprecipitation (ChIP) analysis with an antibody against subunit p65 of NF-κB. Then, specific primers were used in real-time PCR experiments to analyze the *SLC25A1* and *ACLY* gene promoters. (**c**,**d**) Human PBMC-derived macrophages treated as reported above were used to carry out ReChIP experiments with a first antibody against subunit p50 of NF-κB and then with the anti-NF-κB p65 (acetyl K310) antibody. Specific primers were used in real-time PCR experiments to analyze the *SLC25A1* and *ACLY* gene promoters. (**e**) CIC and ACLY protein levels were detected by western blotting in PBMC-derived macrophages treated as above. (**f**,**g**) iBMDM cells transiently cotransfected with the NF-κB (p65) wild-type (NF-κB wt) overexpression vector together with ACLY (p3000) (**f**) or SLC25A1 (p2000) (**g**) promoter vectors and treated with LPS in the presence or absence of SB (**f**,**g**, left panels) or in the presence of ACLY gene silencing (siACLY) (**f**,**g**, right panels), which were used for luciferase luminescence detection. (**h**) iBMDM cells transiently cotransfected with the NF-κB (p65) wild type (NF-κBwt) or mutated (NF-κBmut) and treated with LPS were used to quantify both endogenous ACLY and CIC proteins. Western blotting data presented are representative of at least 3 independent experiments, and the mean of the protein levels quantified against β-actin and normalized versus the mean of the proteins in the untreated cells (C) are reported under the image. In (**a**–**d**,**f**,**g**), data are representative of 3 independent experiments and are presented as means ± SD (error bars). Different letters above the bars indicate significant differences between treatments at *p* < 0.05, according to Tukey’s post hoc test performed after one-way ANOVA.

Since we previously reported active NF-κB responsive elements in both the *SLC25A1* and *ACLY* human gene promoters [37], we hypothesized a functional link between ACLY-mediated NF-κB acetylation and citrate pathway gene transcriptional activation. First of all, chromatin immunoprecipitation with a specific antibody against p65 and in the presence of SB revealed a strong decrease in NF-κB binding to both the *SLC25A1* and *ACLY* gene promoters (Figure 5a,b). Furthermore, Re-ChIP experiments, by using anti-p50 and then anti-AcK310 p65 antibodies, lowered at level of ACLY more than SLC25A1 promoter the increased chromatin immunoprecipitated, as observed upon LPS treatment (Figure 5c,d). In the same experimental conditions, the ACLY and CIC proteins were analyzed. Western blotting experiments showed a reduced amount of both the ACLY and CIC proteins in the presence of an ACLY inhibitor (Figure 5e). These findings suggest a positive ACLY-mediated transcriptional effect on the citrate pathway genes achieved through NF-κB full activation.

Then, we performed cotransfection experiments with an NF-kB (p65) wild type (NF-κBwt) or NF-κB (p65) mutated (K310R) p65 (NF-κBmut) overexpressing construct together with SLC25A1 (p2000) or ACLY (p3000) promoter vectors containing NF-κB-responsive elements upstream of the luciferase gene reporter. Detection of luminescence revealed that the NF-κBwt was able to enhance both the *SLC25A1* and *ACLY* promoter activity in LPS-induced cells, but pretreatment with SB abolished the effect on both promoters (Figure 5f,g, left panels). Conversely, when K310 was mutated, NF-κB overexpression did not affect *SLC25A1* or *ACLY* transcriptional regulation (Figure S4a,b). Interestingly, ACLY gene silencing prevents NF-κB-mediated activation of both the *ACLY* and *SCL25A1* promoters (Figure 5f,g, right panels). To strengthen the relationship between the ACLY/NF-κB axis and transcriptional regulation of both the *SLC25A1* and *ACLY* genes, the endogenous levels of the ACLY and CIC proteins were evaluated after transfection with the NF-κBwt or NF-κBmut overexpressing construct in the presence of LPS. Figure 5h shows a great increase in both the ACLY and CIC proteins when NF-κBwt was used compared with LPS alone. Conversely, the presence of NF-κBmut reverted this effect (Figure 5h).

Moreover, there was not a link between ACLY activation and its transcriptional control through NF-κB when we used a deletion fragment of the ACLY gene promoter (p1000) without the NF-κB-responsive element (Figure S4c,d). Our data indicate that the early ACLY-mediated NF-κB full activation generated a transcriptional upregulation of both genes of the citrate export pathway and triggered the late “hit” of ACLY gene expression in LPS-induced human macrophages.

## 4. Discussion

Recent breakthroughs have highlighted the interplay between metabolism and innate immunity. The growing literature identifies the metabolic shifts as a driving force for immune cells to support their functioning. PAMP-induced M1 macrophages undergo enhanced glycolysis and altered TCA cycles [38,39,40], which in turn produce specific metabolic signals affecting gene expression and cell behavior reprogramming. Citrate is withdrawn from the TCA cycle and exported to the cytosol through the mitochondrial citrate carrier (CIC). Here, its cleavage via ATP citrate lyase (ACLY) is essential to sustain the synthesis of critical inflammatory modulators such as PGE_2_ [41].

In response to LPS, ACLY-derived acetyl CoA is also used for protein malonylation, thus affecting macrophage functions [42]. In further investigating the role of ACLY, our study revealed its timely nuclear translocation in human PAMP-activated macrophages. Here, we demonstrated that ACLY supplies acetyl-CoA for NF-κB subunit p65 acetylation, thus uncovering a critical reason for such early ACLY activation. Our analyses in both LPS- and LTA-activated macrophages indicated ACLY overexpression, as well as its entry into the nucleus and ACLY-mediated p65 acetylation, as features of the macrophage activated by such triggers.

The acetylation at lysine residues 662 and 665 controls ACLY nuclear translocation, since a double mutant construct overexpressing ACLY does not translocate to the nucleus as easily. Lysine acetylation can regulate protein importing across biological membranes [43,44]. Notably, the investigated lysine residues, located at the interface between the cofactor and the substrate binding subunits, are completely accessible for acetylation. Remarkably, the acetyltransferase interacting with ACLY can catalyze K662 and K665 acetylation by using one of the products obtained by ACLY in the conversion of citrate, making the process very efficient. It has also been reported that K540, K546, and K554 (NP_001087 numbering, a shorter isoform) acetylation blocks the ubiquitylation of ACLY, increasing its stability in cancer cells [45]. Therefore, mammalian cells appear to use the same acetyl-CoA produced by ACLY activity for the fine-tuned regulation of different traits of ACLY function. The localization of the investigated lysine residues 662 and 665 far from the cofactor and substrate binding sites should not affect ACLY functions. This is in agreement with previous studies reporting ACLY activity as being controlled by phosphorylation rather than acetylation in murine LPS-induced macrophages [3] and in human atherosclerotic plaques [46].

It is noteworthy that upon ACLY activity inhibition, we found a significant decrease in NF-κB binding to both the *PTGS2* and *IL-1β* gene promoters in human LPS-induced macrophages. In the same experimental conditions, NF-κB-mediated transcriptional activation was reduced. Overall, our data indicate a crucial role of ACLY in driving gene expression by controlling the acetylation status of a master transcription factor of macrophages like NF-κB. Stimulation of the canonical NF-κB pathway is a common event of the PRR-dependent pathways responsible for transcriptional induction of pro-inflammatory genes in M1 macrophages [8]. For this reason, NF-κB activation is a hallmark of countless inflammatory diseases with consequent upregulation of proinflammatory genes [47]. In the synovial membranes and skin samples from patients with rheumatoid arthritis and psoriatic arthritis, respectively, the p65 subunit was expressed, while recently, NF-κB signaling is being investigated for the treatment of rheumatoid arthritis [48,49]. It is noteworthy that the activation of the heterodimer complex p50-p65 seems to be central to the acute respiratory SARS-CoV-2 virus-induced cytokine storm [50,51]. Previous studies pointed out the presence of p65 acetylated following TNFα stimulation [52] and Gram-negative bacterium nontypeable *Haemophilus influenzae* infection [53].

Notably, our investigation revealed ACLY overexpression and p65 acetylation in macrophages differentiated from the PBMCs of septic patients in the early hyperinflammatory phase, when metabolic reprogramming with upregulation of the genes required for glycolysis occurs [54]. Sepsis is also closely related to endotoxemia. In light of the above observations, ACLY inhibition could represent a potential protection against endotoxemia and endotoxic shock that should be further investigated.

It is interesting to consider that, among the specific inhibitors of ACLY, there is also hydroxycitrate (HCA), a natural product which is a main organic acid constituent of the fruit rind of *Garcinia cambogia*. Although its use is limited by its high hydrophilicity and, in turn, its low membrane permeability [41], the existence of an inhibitor already in use as a dietary supplement could more rapidly direct antiseptic and anti-inflammatory drugs to ACLY. HCA fits into the wide range of natural compounds of pivotal importance for current disease treatments [55,56].

Our findings demonstrating the control of NF-κB activity by a metabolic enzyme (ACLY) strengthen the importance of immunometabolism as a new way to look at the regulation of immune cell function. Moreover, by evaluating the promoter activity, we found that ACLY-driven NF-kB acetylation controls the transcription of both the *ACLY* and *SLC25A1* genes. Therefore, ACLY’s rapid activation triggers a positive self-regulated loop inducing full NF-κB activation, which allows citrate pathway exporting by *ACLY* and *SLC25A1* transcriptional upregulation, thus promoting the inflammatory response.

**Figure 6:**
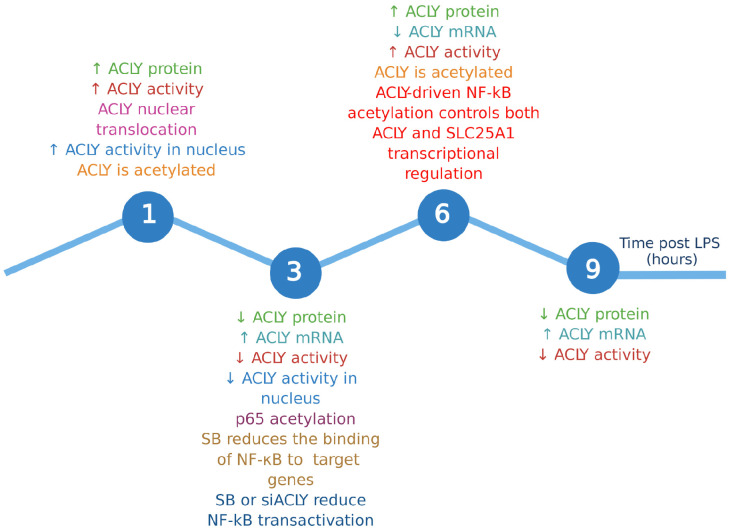
Summary outline showing the timing of the events described in the study. Each event is indicated with a different color. Upward arrows indicate an increase, while downward arrows indicate a reduction to a specific time.

It is likely that the early transcriptionally independent activation of ACLY (Figure 6) may not be immediately functional for lipid biosynthesis (its classical metabolic function). Indeed, ACLY moves to the nucleus to drive gene expression reprogramming—a hallmark of LPS-induced macrophages [57]—by promoting acetylation of both histones, as recently reported [3], and p65. This ACLY-mediated transcriptional control of many proinflammatory genes is needed for macrophage function. Therefore, ACLY can be conceived as a primary enzyme able to divert glucose carbons—derived from enhanced glycolysis after a PAMP addition—toward citrate-derived acetyl-CoA conveyed to the nucleus through ACLY early translocation. Moreover, nuclear ACLY-mediated p65 acetylation significantly concurs with the upregulation of the *SLC25A1* and *ACLY* genes, giving rise to a second peak of ACLY and to the first peak of CIC, which we found expressed later than ACLY previously [17]. In light of these findings, we wonder about the possible function of different citrate pools and the source of the cytosolic citrate. If CIC, exporting citrate from the mitochondria to the cytosol, is not overexpressed shortly during LPS stimulation, where does all the citrate required early in the nucleus for ACLY-mediated acetylation come from? Perhaps it would be interesting to investigate the cellular uptake of citrate triggered by LPS or other PAMPs.

In conclusion, our work provides evidence for two temporally and spatially functional roles of ACLY in PAMP-induced macrophages; its timely nuclear translocation drives NF-κB acetylation and, in turn, gene expression reprogramming with *ACLY* and *SLC25A1* overexpression, whereas its late transcriptional upregulation supports the generation of effector molecules as well as lipids. Both ACLY functions converge toward fostering the immunological phenotype of innate immune cells.

## Figures and Tables

**Table 1:** Characteristics of sepsis patients, showing source of infection and causative pathogens, demographic data (age and sex), and routine blood results from admission of the six sepsis patients enrolled.

Pt	Source of Infection	Age	Sex	WBC (mmc)	Neutrophils (mmc)	CRP (mg/L)	Lactate (mmol/L)	PCT (ng/µL)	PLT (mmc)
1	Uroseptic syndrome with positive blood and urine culture for *E. coli*; multidrug resistant	53	M	18.9	17.5	345 (<5)	4.5 (0.5–2.2)	6 (0–0.5)	140.000
2	Abdominal sepsis caused by perforated diverticulitis; negative blood culture	73	F	21.3	18.5	240 (<5)	4.8 (0.5–2.2)	5 (0–0.5)	170.000
3	BSI (blood stream infection) caused by *Klebsiella pneumoniae* carbapenemase producer (KPC)	78	M	17.4	14.5	85 (<5)	5.1 (0.5–2.2)	14 (0–0.5)	120.000
4	Pyonephrosis with a positive culture for VRE (*Enterococcus faecium* vancomycin resistant) from the nephrostomy sample	59	M	13.4	11.5	157 (<5)	5.1 (0.5–2.2)	10 (0–0.5)	120.000
5	Methicillin-resistant *Staphylococcus aureus* (MRSA) bacteriemia	20	F	18.2	15.5	253 (<5)	5.1 (0.5–2.2)	16 (0–0.5)	190.000
6	Methicillin susceptible *Staphylococcus aureus* (MSSA) prosthetic joint infection	76	M	22.3	18.5	138 (<5)	5.1 (0.5–2.2)	8 (0–0.5)	190.000

Abbreviations: Pt = patient; WBC = white blood cells; CRP = C-reactive protein; PCT = procalcitonin; PLT = platelets; M = male; and F = female. In the brackets, the normal values are reported.

## Data Availability

The data generated during the current study are available from the corresponding author upon reasonable request.

## Supplementary Materials

The following are available online at https://www.mdpi.com/article/10.3390/cells10112962/s1, Figure S1: Mass spectrometry analysis of ACLY acetylation and Evolutionary conservation of ACLY lysine K662 and K665 in Eukarya, Figure S2: Predictions of nuclear localization signals in the ACLY protein sequence, Figure S3: ACLY-derived NF-kB acetylation enhances NF-kB activity, Figure S4: ACLY-derived full NF-kB activity upregulates SLC25A1 and ACLY, Table S1: List of primary antibodies, Table S2: List of constructs and siRNAs.
