# Supplementary material for: ACLY Nuclear Translocation in Human Macrophages Drives Proinflammatory Gene Expression by NF-κB Acetylation
Source: Cells. 2021 Oct 30;10(11):2962. doi: 10.3390/cells10112962 (PMC8616537; doi:10.3390/cells10112962)
Supplement: Supplementary file 1 [file cells-10-02962-s001.zip › cells-1397338-supplementary.pdf]

Supplementary Methods

Multialignment of homologous ACLY protein fragments

ACLY homologous protein sequences were recovered from the HomoloGene database (https://www.ncbi.nlm.nih.gov/homologene/), identifier number 858, were multialigned using Clustal Omega (https://www.ebi.ac.uk/Tools/msa/clustalo/) and graphically represented using Mview (https://www.ebi.ac.uk/Tools/msa/mview/) after duplicate removal.

Predictions of nuclear localization signals

The full sequence of the ACLY/NP_001290203 protein isoform was screened for the presence of nuclear localization signals by using several prediction tools: NLSmapper (http://nls-mapper.iab.keio.ac.jp/cgi-bin/NLS_Mapper_form.cgi), NLStradamus (http://www.moseslab.csb.utoronto.ca/NLStradamus/), NucPred (https://nucpred.bioinfo.se/nucpred/), PSORT II (https://www.genscript.com/psort/psort2.html) and NLSdb (https://rostlab.org/services/nlsdb/).

Cell Viability

Macrophages differentiated in a 96-well plate were used to test cell viability. Cells were incubated with DMSO (control cells) or SB at different concentrations (1 µM, 5 µM and 10 µM) for 48 hours. Evaluation of cell viability was carried out via CellTiter-Glo® 2.0 Cell Viability Assay kit (Promega) following the manufacturer's guidelines. Briefly, 100-µl of CellTiter-glo® 2.0 reagent was added per well and samples were mixed for 2 minutes. To stabilize luminescent signal, the plate was incubated for 15 minutes and then luminescence was measured on a GloMax 96-well luminometer .

Quantification of IL-1β and PGE_2

Human $CD14^+$ macrophages were pre-treated with 5 µM SB-204990 for 1 hour and then triggered by 1 µg/mL of LPS in 24-well plates. Twenty-four hours later, cell-free supernatants were collected and assayed for the concentration of IL-1β cytokine by Luminex100 System (R&D Systems, Inc., Minneapolis, MN, USA) using specific matched-pair antibodies and recombinant cytokines as standards following the manufacturer's recommendations. For PGE_2 quantification cell-free supernatants were collected at the end of 48 hours LPS-treatment. PGE_2 was measured by using DetectX® Prostaglandin E2 High Sensitivity Immunoassay Kit (Arbor Assays, Ann Arbor, MI, USA) following the manufacturers' instructions.

SUPPLEMENTARY FIGURES AND LEGENDS

a

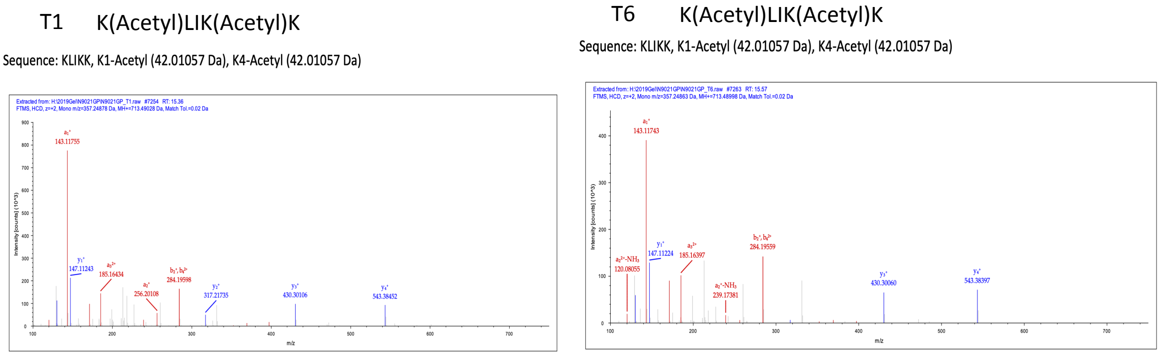

b

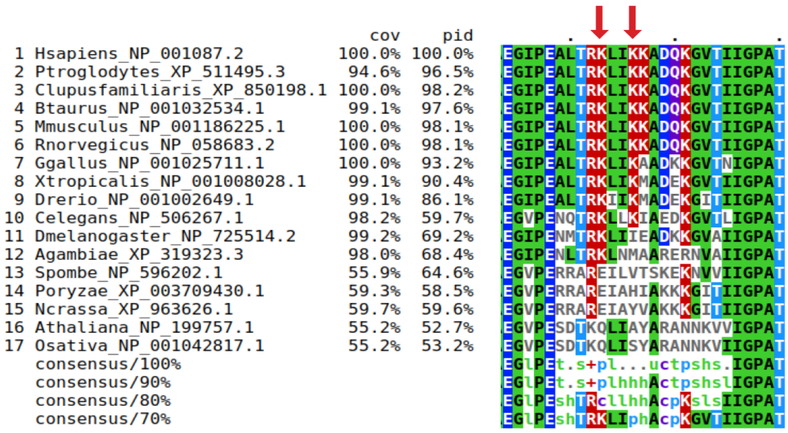

Supplementary Figure S1. Mass spectrometry analysis of ACLY acetylation and Evolutionary conservation of ACLY lysine K662 and K665 in Eukarya. (**a**) MS/MS spectra of acetylated peptides identified in ACLY MS/MS spectra from the acetylated peptides detected on a mass spectrometer following immunoprecipitation of ACLY from PBMC-derived human macrophages. (**b**) Multialignment of seventeen homologous ACLY protein fragments centred on lysine K662 and K665 (red arrows) from representative species of the Eukarya domain. The sequence names indicate the corresponding species and the RefSeq identifier.

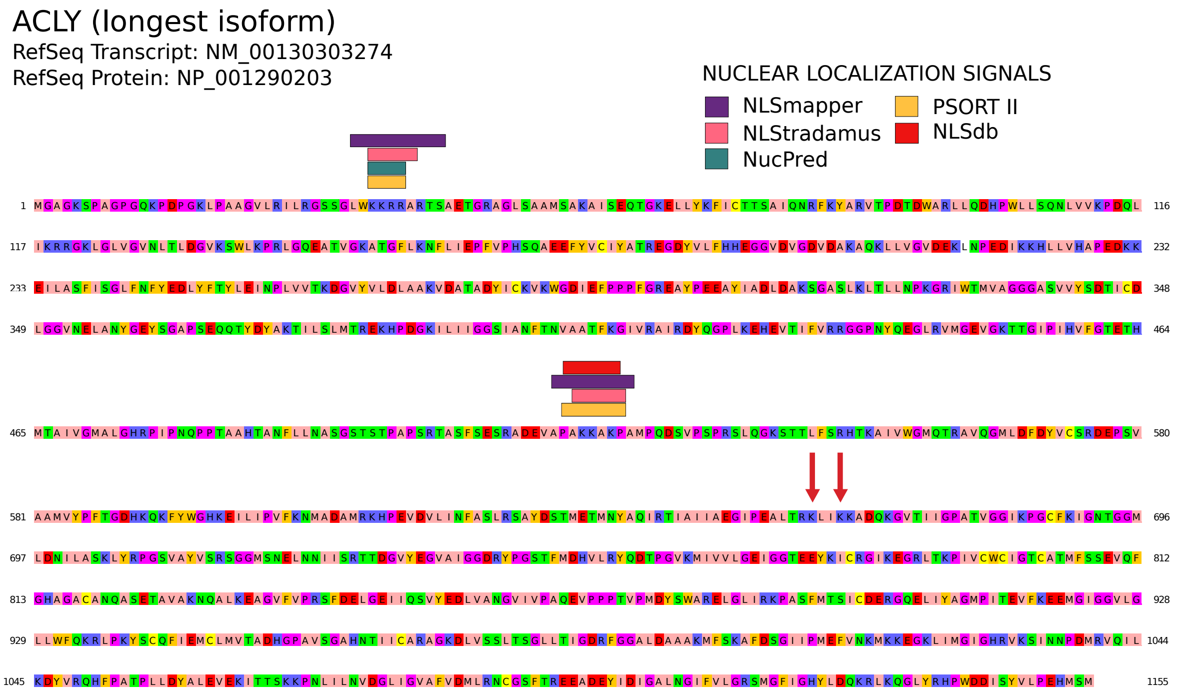

Supplementary Figure S 2. Predictions of nuclear localization signals in the ACLY protein sequence. The full sequence of the longest ACLY protein sequence is reported together with the location of the predicted nuclear localization signals (coloured rectangles above the corresponding peptide). The signal color-code refers to the name of the software that predicted each signal. Red arrows indicate lysine K662 and K665.

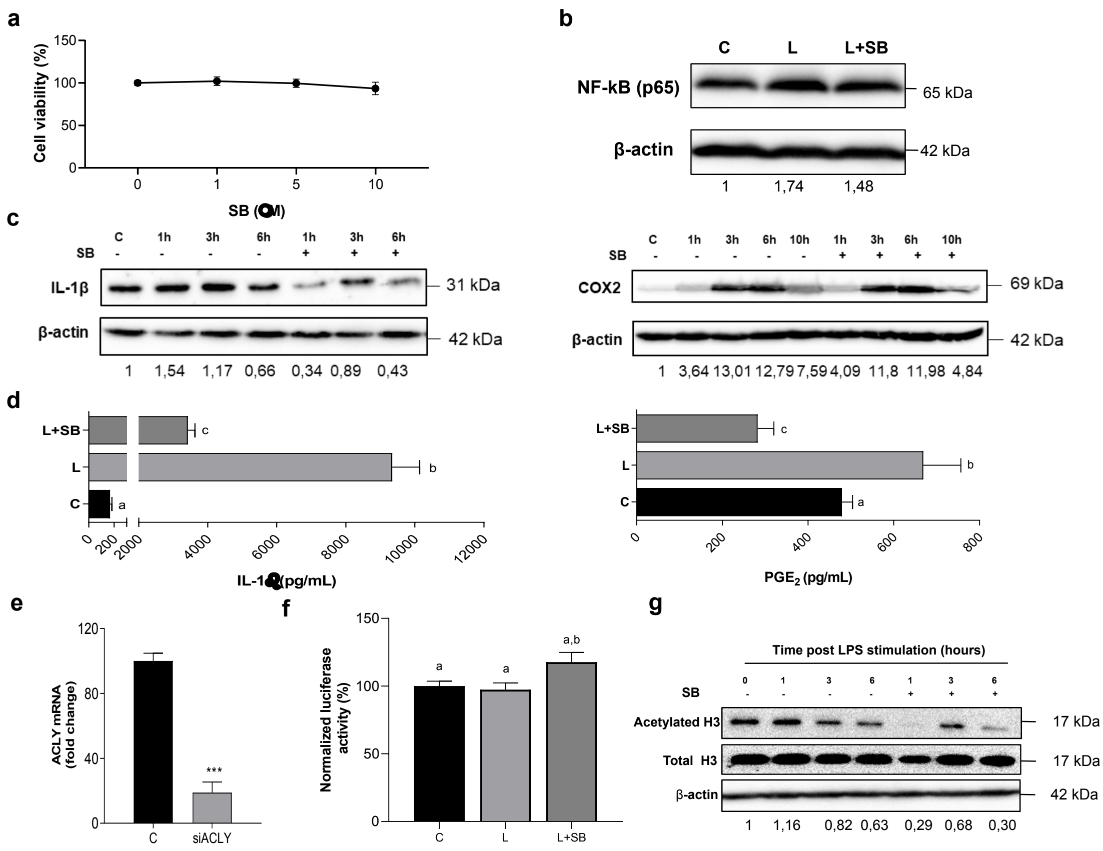

Supplementary Figure S3. ACLY-derived NF-kB acetylation enhances NF-kB activity (**a**) Cell viability of human PBMC-derived macrophages untreated or treated for 48 h with 1μM, 5μM or 10μM SB-204990 (SB) was evaluates. (**b**) Human PBMC-derived macrophages treated with LPS for 3 h in the presence or not of SB-204990 (SB) were lysed and used to quantify NF-kB (p65) protein levels. (**c**) Human PBMC-derived macrophages were treated with LPS for 3 h in the presence or not of SB-204990 (SB) were lysed and used to quantify IL-1β and COX2 protein levels. (**d**) IL-1β and PGE_2 secretion was measured in LPS-treated human macrophages in the presence or absence of SB. (**e**) iBMDM were transfected with siRNA targeting human ACLY (siACLY) or control scramble siRNA and used to quantify ACLY mRNA (**f**) iBMDM transiently cotransfected with ACLY P_{mut} overexpressing construct together with NF-kB luc promoter vector were used for luciferase luminescence detection (**g**) Human PBMC-derived macrophages treated as in (**a**) were used to quantify H3 acetylated histone. In (**a**) and (**e**) statistical significance of differences was evaluated by using Student's t-test. In (**d**) and (**f**) According to one-way ANOVA, differences were significant. Therefore, Tukey's post hoc test was performed and different letters indicate significant differences between treatments at p<0.05. Western blotting data presented are representative of at least 3 independent experiments. In (**b**) and (**c**), protein levels quantified against β-actin and normalized versus untreated cells (C) are reported under western blotting images. In (**g**), acetylated H3 levels quantified against total H3 and normalized versus untreated cells (0) are reported under the image.

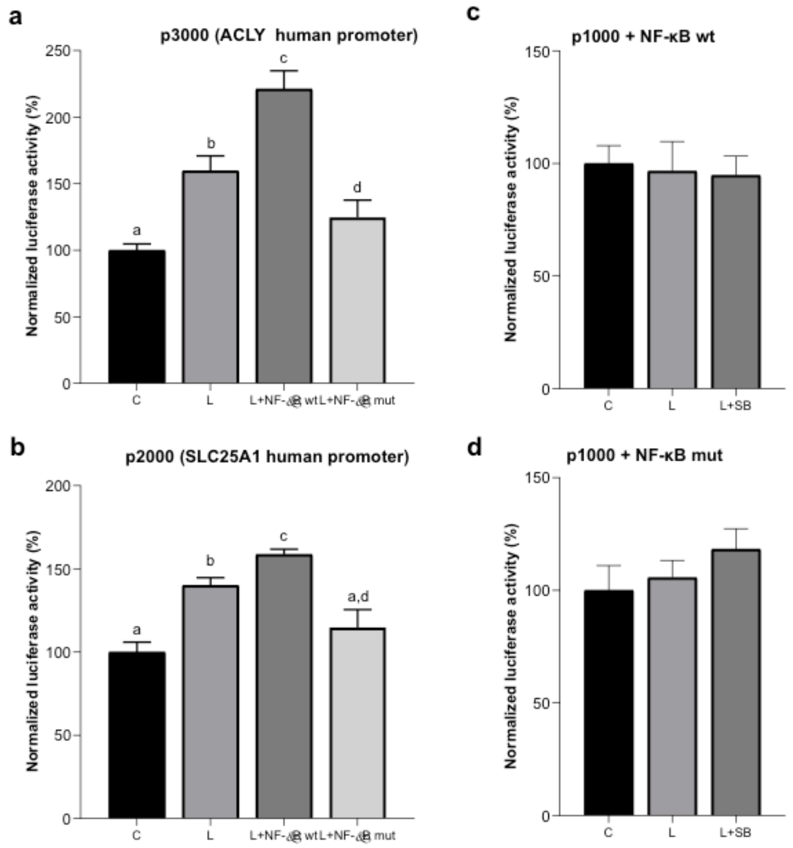

Supplementary Figure S4. ACLY-derived full NF-kB activity upregulates *SLC25A1* and *ACLY* (**a**) and (**b**) Luciferase activity was quantified in iBMDM transiently cotransfected with NF-kB (p65) wild-type (NF-kB wt) or NF-kB (p65) mutated (K310R) p65 (NF-kB mut) overexpressing construct together with ACLY (p3000) (**a**) or SLC25A1 (p2000) (**b**) promoter vector and treated with LPS. (**c**) and (**d**) iBMDM transiently cotransfected with NF-kB (p65) wild-type (NF-kB wt) (**c**) or NF-kB (p65) mutated (K310R) p65 (NF-kB mut) (**d**) overexpressing construct together with SLC25A1 (1000) promoter vector and treated with LPS in the presence or absence of SB were used for luciferase luminescence detection. Data are representative of three independent experiments and are presented as means ± SD. In (**a**) and (**b**), according to one-way ANOVA, differences were significant. Therefore, Tukey's post hoc test was performed and different letters indicate significant differences between treatments at $p<0.05$. In (**c**) and (**d**), according to one-way ANOVA, differences were not significant.

Table S1. List of primary antibodies.

Antigen	Host species	Source	Cat. No
ATP-citrate lyase	Rabbit	Abcam	ab157098
NF-κB p65	Rabbit	Abcam	ab7970
Histone H3 (acetyl K9 + K14 + K18 + K23 + K27)	Rabbit	Abcam	ab47915
Histone H3	Rabbit	Abcam	ab1791
IL-1β	Rabbit	Abcam	ab226918
Cyclooxygenase 2	Rabbit	Abcam	ab15191
Acetylated-Lysine	Rabbit	Cell Signaling Technology	9441S
NF-kB p65 (acetyl K310)	Rabbit	Abcam	ab19870
Lamin A + Lamin C	Rabbit	Abcam	ab58529
Citrate carrier	Rabbit	Thermo Fisher Scientific	PA5-42451
β–actin	Rabbit	Abcam	ab8227
DDDDK tag	Rabbit	Abcam	ab205606

Table S2. List of constructs and siRNAs.

Construct	Source	
ACLY wild-type (Pwt)	Genscript Biotech	**ID**: ACLY_OHu50424D_pcDNA3.1+/C-(K)-DYK
ACLY mutant (Pmut)	Genscript Biotech	**ID**: ACLY_OHu50424D_Mutant1_pcDNA3.1+/C-(K)-DYK
pGL3–5xNF-κB	Promega	**Cat. No**: E8491
SLC25A1pGL3		[1]
ACLY gene promoter (3000)		[2]
ACLY gene promoter (1000)		[2]
pRL-CMV	Promega	**Cat. No**: E2261
NF-κB p65 subunit wild-type (NF-κB wt)	Genscript Biotech	**ID**: RELA_OHu26911D_pcDNA3.1+/C-(K)-DYK
NF-κB p65 subunit K310R mutated (NF-κB mut)	Genscript Biotech	**ID**: RELA_OHu26911D_K310R_pcDNA3.1+/C-(K)-DYK
siRNA targeting human ACLY	Thermo Fisher Scientifc	**ID:** s915
Silencer™ Select Negative Control	Thermo Fisher Scientifc	**Cat. No:** 4390843

[1] Infantino V.; Convertini P.; Cucci L.; Panaro M.A.; Di Noia M.A.; Calvello R.; Palmieri F.; Iacobazzi V. ACCELERATED PUBLICATION The mitochondrial citrate carrier: a new player in inflammation. *Biochemical Journal* 2011, *438*, 433-436.
[2] Santarsiero A.; Onzo A.; Pascale R.; Acquavia M.A.; Coviello M.; Convertini P.; Todisco S.; Marsico M.; Pifano C.; Iannece P. and others. Hydrosol: Untargeted Metabolomic Analysis and Anti-Inflammatory Activity Mediated by NF-. *Oxid Med Cell Longev* **2020**, *2020*, 4264815.
